# High-throughput screening for agonists of ROS production in live human vascular endothelial cells

**DOI:** 10.1016/j.xpro.2021.101053

**Published:** 2021-12-21

**Authors:** Tomoya Sasahara, Minako Hoshi

**Affiliations:** 1 Department for Brain and Neurodegenerative Disease Research, Institute of Biomedical Research and Innovation, Foundation for Biomedical Research and Innovation at Kobe, CLIK 6F 6-3-7 Minatojima-Minamimachi, Chuo-ku, Kobe 650-0047, Japan; 2 TAO Health Life Pharma Co., Ltd., Med-Pharma Collaboration Bldg, Kyoto University, 46-29 Yoshida Shimoadachi-cho, Sakyo-ku, Kyoto 606-8501, Japan; 3 Department of Anatomy and Developmental Biology, Graduate School of Medicine, Kyoto University, Kyoto 606-8501, Japan

**Keywords:** Cell Biology, Cell-based Assays, High Throughput Screening, Microscopy, Molecular/Chemical Probes

## Abstract

Reactive oxygen species (ROS) are important physiological molecules, and identifying agonists for ROS production can yield useful tools for future research. Here we present an optimized protocol for high-throughput screening for agonists that induce ROS production. We describe the use of a fluorescent probe in human vascular endothelial cells, which can establish whether ROS production occurs in mitochondria or in the plasma membrane of live cells.

For complete details on the use and execution of this profile, please refer to Sasahara et al. (2021).

## Highlights

• This is a high-throughput screening protocol for agonists that induce ROS production
• ROS production in human endothelial cells is identified by fluorescence imaging
• This reveals whether ROS production occurs in mitochondria or plasma membrane

## Graphical abstract

**Figure:**
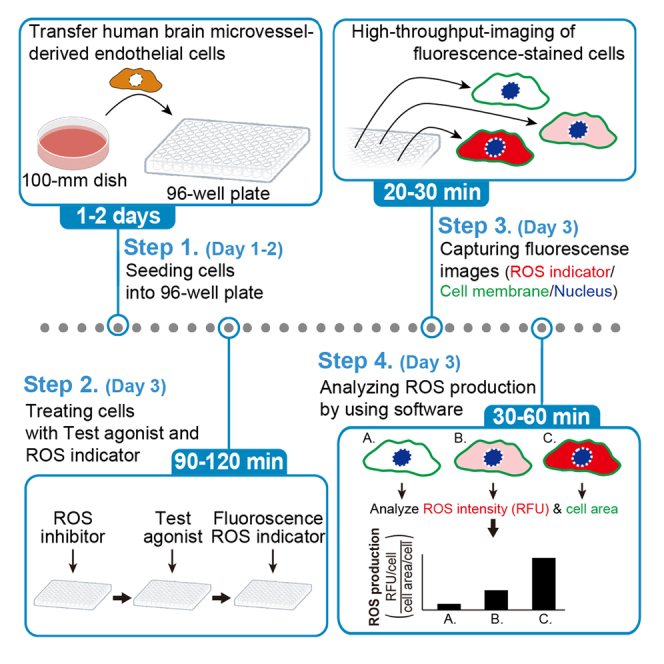

## Before you begin

Before starting the protocol, prepare the culture of vascular endothelial cells and stock solutions of fluorescent cell membrane marker, reactive oxygen species (ROS) inhibitors, and lipopolysaccharide (LPS).

### Cell culture: Coating the bottom of the culture plate with collagen-I

**Timing: 3 h**

***Note:*** The steps 1-b and 2 must be performed on a clean bench.

The protocol below describes the steps for coating the bottom of the culture plate with collagen-I.

1. Prepare distilled water adjusted to pH 3.0.
  a. Adjust the pH of distilled water to 3.0 by using a pH meter and HCl.
  b. Sterilize the distilled water adjusted to pH 3.0 using a PVDF membrane filter with a 0.22 μm pore size.
2. Coat the bottom of the plate with collagen-I.
  a. Dilute collagen-I ten-fold in distilled water adjusted to pH 3.0.
  b. Add 3 mL and 40 μL of the diluted collagen-I to a 100-mm culture dish and to each well of a 96-well culture plate (flat and clear bottom; black side), respectively.
  c. Incubate for 1 h at 22°C–25°C.
  d. Rinse the dish and the plate three times with phosphate-buffered saline without calcium and magnesium (PBS(–)).
  e. Add 8 mL and 50 μL PBS(–) into the dish and each well, respectively, and store at 4°C until use.

***Note:*** The collagen-I-coated culture dish and culture plate should be used within 24 h.

### Cell culture: Thawing and maintaining vascular endothelial cells

**Timing: 1 week**

The protocol below describes the steps for thawing primary human brain microvessel endothelial cells and maintaining the cells.

***Note:*** The endothelial cells utilized in the present protocol have not been designated as BSL because no adverse events have been reported so far. However, we recommend BSL-2 compliance just in case. The steps 3–5 must be performed on a BSL-2 safety cabinet.

***Note:*** In all steps, the EGM2-MV culture medium and PBS(–) should be warmed to 37°C before use to avoid cold stimulation of the cells.

3. Thaw the cells in a cryovial.
  a. Place the frozen vial of cells in a water bath at 37°C for 1 min.
  b. To prevent contamination, spray the vial with 70% ethanol and wipe it off carefully.
  c. Transfer the solution in the vial to a 50 mL centrifuge tube containing 9 mL of the medium.
4. Seed the cells in a 100-mm culture dish.
  a. Centrifuge the cells at 450×*g* for 5 min.
  b. Discard the supernatant.
  c. Suspend the cells in 10 mL of the medium.
  d. Count the cells in the suspension.
  e. Discard PBS(–) from the collagen-I-coated 100-mm culture dish prepared in step 1.
  f. Seed approximately 1 × 10^6^ cells into the dish.
  g. Culture the cells in the dish with 8 mL of the medium.
  h. Incubate at 37°C under 5% CO_2_ (Do not disturb the cells for at least 1 h after seeding to avoid affecting cell adhesion).
5. Maintain the cells.
  a. Change the medium every 2–3 days.
  b. Passage the cells when the cells reach 70%–80% confluence (the subculture ratio is 1:4).

**CRITICAL:** To avoid loss of endothelial cell-specific properties, the cells should be passaged at 70%–80% confluency, and culture should not be continued for more than 8 passages.

### Stock solution: Preparing fluorescent cell membrane marker

**Timing: 30 min**

The protocol below describes the steps for preparing the stock solution of fluorescent cell membrane marker, wheat germ agglutinin Alexa Fluor 488 (WGA AF488) conjugate.

***Note:*** Step 6 must be performed on a clean bench.

6. Prepare a stock of WGA AF488 conjugate (see also Table 1).
  a. Dissolve WGA AF488 conjugate in PBS(–) to 1 mg/mL.
  b. Store at ≤−20°C with protection from light.

**Table 1:** List of reagents to be prepared

Reagent	Target of reagent	Stock conc.	Stock preparation (powder:solvent)	Storage of stock solutions	Working conc.
WGA AF488	fluorescent cell membrane marker	1 mg/mL	1 mg:1 mL PBS(–)	≤−20°C, for up to 1 month with protection from light	0.01 mg/mL
N-acetyl-L-cysteine	non-specific ROS scavenge	250 mM	1 mg:24.5 μL PBS(–)	≤−20°C, for up to 1 month	500 μM
mito-tempol	mitochondrial ROS-specific inhibition	50 mM	1 mg:34.3 μL PBS(–)	≤−20°C, for up to 1–2 weeks	100 μM
YCG-063	mitochondrial ROS-specific inhibition	25 mM	1 mg: 95.4 μL DMSO	≤−20°C, for up to 1–2 weeks	50 μM
VAS2870	NADPH oxidase-specific inhibition	5 mM	1 mg:554.9 μL DMSO	≤−20°C, for up to 6 months	10 μM
apocynin	NADPH oxidase-specific inhibition	10 mM	1 mg:601.7 μL DMSO	≤−20°C, for up to 6 months	20 μM
LPS	ROS agonist	1–5 mg/mL	1–5 mg:1 mL PBS(–)	−80°C, for up to 3 months	1 μg/mL

conc., concentration.

### Stock solution: Preparing ROS inhibitors

**Timing: 1–2 h**

The protocol below describes the steps for preparing stock solutions of ROS inhibitors.

The selectivity and stock concentrations of the inhibitors are shown in Table 1. Troubleshooting 1

***Note:*** The steps 7–8 must be performed on a clean bench.

7. Prepare the stock solutions of YCG-063, VAS2870, and apocynin (the proportions of powder:solvent are shown in Table 1).
  a. Dissolve YCG-063, VAS2870, and apocynin in DMSO.
  b. Store at ≤−20°C.

***Note:*** DMSO is cytotoxic at concentrations of 0.2% or higher. Therefore, we recommend you prepare stock solutions at a concentration more than 500 times higher than the final working concentration.

8. Prepare stock solutions of N-acetyl-L-cysteine and mito-tempol (the proportions of powder:solvent are shown in Table 1).
  a. Dissolve N-acetyl-L-cysteine and mito-tempol in PBS(–).
  b. Store at ≤−20°C.

### Stock solution: Preparing LPS

**Timing: 30 min**

The protocol below describes the steps for preparing the LPS stock solution.

***Note:*** Step 9 must be performed on a clean bench.

***Note:*** LPS is utilized as a positive control agonist of ROS production.

9. Prepare LPS stock solution (see also Table 1).
  a. Dissolve LPS in PBS(–) to 1–5 mg/mL. Troubleshooting 2
  b. Store at −80°C.

## Key resources table

**Table:** 

REAGENT or RESOURCE	SOURCE	IDENTIFIER
**Chemicals, peptides, and recombinant proteins**		

DMSO (non-fluorescent)	FUJIFILM Wako	Cat# 344-03611
Collagen-I	Nitta Gelatin	Cat# Cellmatrix Type I-C
EGM2-MV culture medium	Lonza	Cat# CC-3202
Phosphate-buffered saline without calcium and magnesium (PBS(–)), pH 7.4	Nacalai Tesque	Cat# 13397-85
Hanks’ balanced salt solution containing calcium and magnesium (HBSS(+))	Nacalai Tesque	Cat# 09735-75
0.25% Trypsin including 1 mM EDTA	Nacalai Tesque	Cat# 35554-64
Wheat germ agglutinin Alexa Fluor 488 conjugate	Thermo Fisher Scientific	Cat# W11261
N-acetyl-L-cysteine	Merck Millipore	Cat# 106425
mito-tempol	Cayman Chemical	Cat# 18796
YCG-063	Merck Millipore	Cat# 557354
VAS2870	Merck Millipore	Cat# 492000
Apocynin	Merck Millipore	Cat# 178385
Lipopolysaccharide	Sigma-Aldrich	Cat# L2630
CellROX orange	Thermo Fisher Scientific	Cat# C10443
Hoechst 33342	Dojindo Molecular Technologies	Cat# H342

**Experimental models: Cell lines**		

Human: primary human endothelial cells derived from brain microvessels (isolated from normal healthy donor tissues (details of the donors including gender are not available))	Cell Systems	Cat# ACBRI 376

**Software and algorithms**		

CQ1 software (Ver. 1.06.01.04)	Yokogawa Electric Corp.	https://www.yokogawa.co.jp/library/documents-downloads/software/lsc-cq1-software/

**Other**		

96-well culture plate (flat and clear bottom; black side)	Falcon	Cat# 353219
100-mm culture dish	Falcon	Cat# 353003
PVDF membrane filter with a 0.22 μm pore size	Merck Millipore	Cat# SLGVR33RS
High-content analysis CQ1	Yokogawa Electric Corp.	N/A

## Materials and equipment

**Table:** Mixture of of CellROX orange, WGA AF488 conjugate, and Hoechst 33342

Reagent	Concentration in mixture	Preparation of mixture	Working concentration (1/50 of mixture)
CellROX orange (2.5 mM)	250 μM	50 μL	5 μM
WGA AF488 conjugate (1 mg/mL)	0.5 mg/mL	250 μL	0.01 mg/mL
Hoechst 33342 (1 mg/mL)	100 μg/mL	50 μL	2 μg/mL
PBS(–)	N/A	150 μL	N/A
Total	N/A	500 μL	N/A

Prepare just before use in step 8 (step-by-step method details). Store at 4°C .

***Alternatives:*** This protocol for measuring the amount of ROS production in live cells requires a device that can capture multiple fluorescence staining images at a very high speed. To our knowledge, CQ1 currently offers the fastest image capture, and therefore, we recommend you use CQ1. However, other high-content analysis devices, such as ArrayScan (Thermo Fisher Scientific) and ImageXpress Micro Confocal (Molecular devices), or fluorescence microscopy can be used. See details in troubleshooting 4.

## Step-by-step method details

**Pause Point:** There are no pause points during the three days of the experiment. Before you start, please secure enough time for the experiment.

### Prepare cultured vascular endothelial cells for measurement of ROS production

**Timing: (Day 1–2) 1–2 h at Day 1, ∼1 h at Day 2.**

In this section, we describe the method for seeding endothelial cells from a 100-mm culture dish into a 96-well culture plate for the measurement of ROS production.

***Note:*** The steps 1–3 must be performed on a BSL-2 safety cabinet.

***Note:*** In all steps, EGM2-MV medium and PBS(–) should be warmed to 37°C before use to avoid cold stimulation of the cells.

1. (Day 1) Collect the cells from a 100-mm culture dish.
  a. Discard the medium.
  b. Rinse the cells with PBS(–).
  c. Discard the PBS(–).
  d. Add 3 mL of 0.25% trypsin solution with EDTA.
  e. Incubate at 37°C for 2–3 min.
  f. Add 7 mL of the medium.
  g. Strip the cells from the dish by tapping the side of the dish twice and gently pipetting.
  h. Transfer the cells into a 50 mL centrifuge tube.
2. (Day 1) Seed the cells in a 96-well culture plate.
  a. Centrifuge the cells at 450×*g* for 5 min.
  b. Discard the supernatant.
  c. Suspend the cells in the medium.
  d. Count the cells in the suspension.
  e. Discard PBS(–) from the collagen-I-coated 96-well culture plate (flat and clear bottom; black side) prepared in step 1 (“before you begin”).
  f. Seed approximately 1.0 × 10^4^ cells into each well of the plate.
  g. Culture the cells in each well of the plate with 200 μL of the medium.
  h. Incubate at 37°C under 5% CO_2_ for at least 16 h (Do not disturb the cells for at least 1 h after seeding to avoid affecting cell adhesion).

***Note:*** The outermost wells of the plate (rows A and H, columns 1 and 12) cannot be measured by the high-content analysis CQ1, and therefore, you can use only the 60 wells defined by rows B-G and columns 2–11. Troubleshooting 3

3. (Day 2) Change the medium.
  a. Discard the medium.
  b. Add fresh medium.
  c. Incubate at 37°C under 5% CO_2_ for at least 16 h.

### Set up the measurement protocol of the high-content analysis CQ1

**Timing: (Day 3) ∼30 min**

In this section, we describe the method for setting up the measurement protocol in the CQ1 software to capture multiple fluorescence images of CellROX orange staining, WGA AF488 conjugate staining, and Hoechst 33342 staining. Troubleshooting 4

4. Set up the measurement protocol of CQ1 software as follows.
  a. To capture Hoechst 33342 staining, set a combination of 405 nm excitation wavelength and emission filter 447/60 in channel 1.
  b. To capture WGA AF488 conjugate staining, set a combination of 488 nm excitation wavelength and emission filter 525/50 in channel 2.
  c. To capture CellROX orange staining, set a combination of 561 nm excitation wavelength and emission filter 617/73 in channel 3.
  d. Set the minimum exposure time of all channels.
  e. Set the binning number of image resolution to 8.
  f. Set 5 fields of view in a well.
  g. Set a normal-focus 20× magnification lens.
  h. Set the Z stack condition to 5 slice images with 3 μm steps.

**CRITICAL:** Steps d and e are important to minimize the difference in measurement time between the first- and the last-measured wells.

***Note:*** In our experience, these settings in steps f and g enable analysis of approximately 500 cells in one well.

### Treat endothelial cells with test agonist, and load the cells with ROS fluorescence probe

**Timing: (Day 3) 90–120 min**

In this section, we describe the method for pretreating human vascular endothelial cells on a 96-well culture plate with ROS inhibitors, treating the cells with test reagents (hereafter termed Test agonists), and loading the cells with the ROS fluorescence probe.

***Note:*** The steps 5–8 must be performed on a BSL-2 safety cabinet.

***Note:*** Before starting the protocol, thaw the stock reagents prepared in “before you begin” (WGA AF488 conjugate, ROS inhibitors, and LPS) and the commercial products (CellROX orange and Hoechst 33342).

5. Pretreat the cells with ROS inhibitors.
  a. Treat cells with each ROS inhibitor (working concentrations of inhibitors are 500-fold dilution of the stock concentration (see Table 1)).
  b. Incubate for 30 min at 37°C under 5% CO_2_.

***Note:*** If dilution of the inhibitors is required, dilute with PBS(–).

6. Treat the cells with Test agonists.
  a. Treat cells with each Test agonist.
  b. Incubate for the appropriate time at 37°C under 5% CO_2_.
7. 30 min before the end of incubation, treat the cells for the positive control group with LPS (1 μg/mL).
8. Load the cells with ROS fluorescence probe.
  a. Immediately after step 7, treat all cells with a mixture of CellROX orange (5 μM, an ROS fluorescence probe), WGA AF488 conjugate (0.01 mg/mL, a fluorescent cell membrane counterstain), and Hoechst 33342 (2 μg/mL, a nuclear counterstain) (the preparation of a mixture is shown in “materials and equipment”).
  b. Incubate for 30 min at 37°C under 5% CO_2_.

### Capturing fluorescence images with CQ1

**Timing: (Day 3) ∼30 min**

In this section, we describe the method for capturing multiple fluorescence images of CellROX orange staining, WGA AF488 conjugate staining, and Hoechst 33342 staining by using CQ1.

***Note:*** The step 9 must be performed on a BSL-2 safety cabinet.

***Note:*** Hanks’ balanced salt solution containing calcium and magnesium (HBSS(+)) and EGM2-MV medium should be warmed to 37°C before use to avoid cold stimulation of the cells.

9. Wash the cells.
  a. Discard the medium from all wells.
  b. Wash the cells twice with HBSS(+).
  c. Discard HBSS(+) from all wells.
  d. Add fresh medium to all wells.

***Note:*** To avoid stress stimulation of cells due to the use of low-nutrient medium, this protocol utilizes a complete medium, EGM2-MV. The use of EGM2-MV has little effect on the background.

10. Place the 96-well culture plate into CQ1. Troubleshooting 5
11. In the pre-recording mode, set the power of the excitation lasers for channel 1, channel 2, and channel 3 to the appropriate intensity to obtain fluorescence images.
12. Capture the fluorescence images.

### Analysis of ROS production with CQ1

**Timing: (Day 3 or later) ∼60 min**

In this section, we describe a method for analyzing the amount of CellROX orange-derived ROS production per cell area from the fluorescence images captured by CQ1.

13. Create the analysis protocol on CQ1 software as follows (Figure 1).
  a. Create an analysis protocol (termed “Object 1”) to detect cell nuclei from the maximum intensity projection (MIP) image of Hoechst 33342 staining (as shown in the center image of Figure 1).
  b. Create an analysis protocol (termed “Object 2”) to detect the whole cell area of endothelial cells, including one nucleus detected in step a, from the MIP images of WGA AF488 conjugate staining (as shown in the right image of Figure 1).

**Figure 1:**
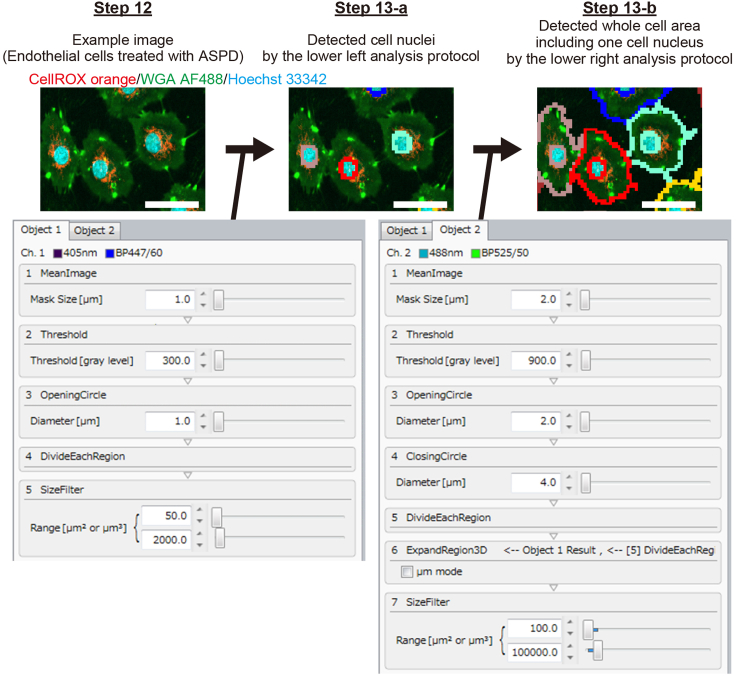
Detection of the cell nucleus and cell membrane with CQ1 software See step 13. The upper left image shows endothelial cells treated with Aβ assemblies for 6 h, amylospheroids (ASPD, 35 nM; this concentration is sufficient to induce ROS production in mitochondria (Sasahara et al., 2021)). By analyzing the upper left image with the protocol shown in the lower left, the cell nuclei are detected (see the upper middle image) (the periphery of each nucleus is traced in a different color). Then, by analyzing the upper middle image with the protocol shown in the lower right, the whole cell area of each cell, in the center of which the detected nucleus is present, is obtained (see the upper right image) (the periphery of each cell area is traced in the same color as that of the nucleus).
14. Analyze the MIP images of fluorescence staining captured in step 12 using the analysis protocol created in step 13.
15. Extract the following values from the analysis results exported as an Excel file.
  a. Extract the value of total fluorescence intensity of CellROX staining, which represents ROS production per cell, from the column titled "(Object 2) TotalIntensity CH3”.
  b. Extract the value of the WGA AF488 conjugate staining area, which represents cell area per cell, from the column titled "(Object 2) area”.
  c. If necessary, extract the value of the number of Hoechst 33342 stainings, which represents the cell number analyzed, from the column titled "Count”.
16. Calculate the value of ROS production per cell area from the values.
  a. Divide [the value of total fluorescence intensity of CellROX staining per cell (obtained in step 15-a)] by [the value of WGA AF488 conjugate-staining area per cell (obtained in step 15-b)].

## Expected outcomes

**Figure 2:**
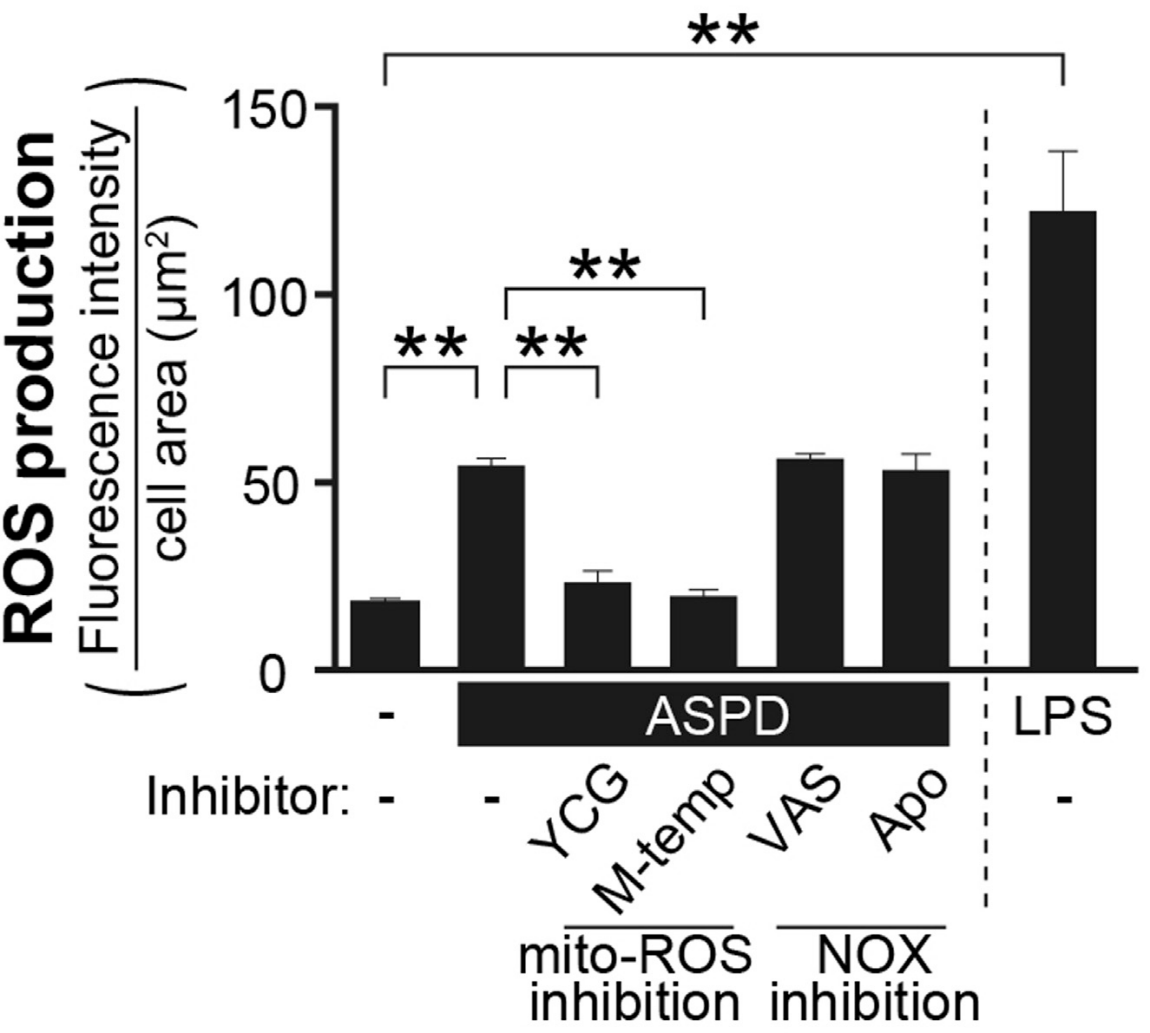
Typical results obtained by means of this protocol An example of results obtained using this protocol is shown (Sasahara et al., 2021) (the vertical axis was modified from % to the value of CellROX orange-derived fluorescence intensity per cell area, and the values of the non-treated group and LSP-treated group were included). The endothelial cells were pretreated with inhibitors of mitochondrial ROS (mito-ROS: YCG-063 (YCG, 50 μM) and mito-tempol (M-temp, 100 μM)) or of NADPH oxidase (NOX: VAS2870 (VAS, 10 μM) and apocynin (Apo, 20 μM)) for 30 min, and then further treated with Aβ assemblies, amylospheroids (ASPD, 35 nM), for 6 h. As a positive control of ROS production, the endothelial cells were treated with LPS (0.1 μg/mL) for 0.5 h. ROS production was estimated by monitoring the fluorescence intensity of a ROS fluorescence probe, CellROX orange, with CQ1 (n = 4). YCG-063 and mito-tempol blocked the ROS production induced by ASPD. This suggests that the increase of ROS production by ASPD occurs in mitochondria. Data are presented as means ± S.E. ∗∗*P* < 0.01 (ANOVA with Scheffé’s method calculated by Statcel2 software).

You can determine which Test agonists increase ROS production in human brain microvessel-derived endothelial cells (an example is shown in Figure 2). By pretreating the cells with ROS inhibitors, you can determine whether the increase of ROS production induced by each Test agonist occurs in mitochondria, plasma membrane (mediated by NADPH oxidase), or other locations (Figure 2). In the protocol provided here, all processes from cell imaging to quantification are automated by the high-content analysis CQ1, and thereby, it is possible to obtain more objective data excluding observer bias in visual field selection and analysis. Because the image analysis with CQ1 software can selectively analyze the intracellular areas, the protocol can quantify ROS-derived fluorescence staining in the cells while excluding non-specific staining derived from extracellular debris. This protocol allows us to screen agonists potentially relevant to vascular diseases such as hypertension, atherosclerosis, hyperlipidemia, diabetes, and Alzheimer’s disease-associated vascular dysfunction, which are associated with the increase of ROS production in endothelial cells (Brieger et al., 2012; Singh et al., 2019).

We believe that our protocol would also be applicable for other purposes with some modifications. Here we show two examples, as follows. 1) Screening for ROS inhibitor candidates can be done using a modification of this protocol, in which cells are pretreated with ROS inhibitor candidates and then treated with a ROS-producing agonist. 2) The protocol can be used to evaluate whether various agonists increase ROS production in adhesive cells other than endothelial cells.

## Limitations

This protocol can establish whether ROS production induced by Test agonist occurs in mitochondria or in plasma membrane. However, the involvement of other ROS-producing enzymes, such as peroxisome, xanthine oxidase, NO synthase, P450 cytochromes, etc. (Brieger et al., 2012), cannot be clarified. Because CellROX orange is not compatible with cell fixation, ROS production should be measured in live cells. Therefore, we recommend the use of the high content analysis CQ1, because to our knowledge it offers the fastest image capture among currently available high-content analysis devices (see details in troubleshooting 4).

## Troubleshooting

### Problem 1

We examined the effects of the typical non-selective ROS scavengers, N-acetyl-L-cysteine and tempol. N-Acetyl-L-cysteine completely suppressed the ROS-derived increase of fluorescence intensity, while tempol did not. Why? (Steps 7 and 8 in before you begin)

### Potential solution

In the process of establishing this protocol, we found that tempol is itself fluorescent. Because this protocol evaluates ROS production by analyzing fluorescence images, the use of reagents with strong intrinsic fluorescence, such as tempol, is unsuitable. In the fluorescence images of cells treated with tempol alone, you will see that non-negligible levels of strong fluorescence spread throughout the cytoplasm of the tempol-treated cells. This autofluorescent derived from tempol should interfere with the analysis of fluorescence images. If you intend to use a ROS inhibitor that is not listed in this protocol, we recommend you first make sure that the inhibitor is not itself fluorescent.

In our protocol, we utilize a mitochondria-targeting tempol analog, mito-tempol, which is also fluorescent. However, its fluorescence background is not high enough to affect the experimental results. This may be because mito-tempol is specifically localized to mitochondria, in contrast to tempol, which is non-specifically localized in the cytoplasm, and the optimal concentration of mito-tempol (100 μM) is much lower than that of tempol (3 mM in general).

### Problem 2

The prepared stock of LPS rapidly deactivates. (Step 9 in before you begin)

### Potential solution

Have you created a stock solution of a lower or higher concentration than the indicated value? To our knowledge, the optimal concentration of LPS stock solution is 1–5 mg/mL. Stock solutions of lower concentration are less stable, while more concentrated stock solutions lead to the formation of LPS aggregates.

### Problem 3

The outermost wells of the plate cannot be measured with CQ1. However, we want to measure all wells. (Step 2 in step-by-step method details)

### Potential solution

In our protocol, we use a normal-focus 20× magnification lens to capture images. If a long-focus 20× magnification lens is mounted on the CQ1, you can measure all 96 wells.

### Problem 4

High-content analysis CQ1 is not available in the laboratory. Are there alternative methods? (Step 4 in step-by-step method details)

### Potential solution

This protocol for measuring the amount of ROS production in live cells requires a device that can capture multiple fluorescence staining images at a very high speed. To our knowledge, CQ1 currently offers the fastest image capture. However, if other high-content analysis devices can measure a 96-well culture plate within 15 min, they may be suitable as a substitute for CQ1. If no high-content analysis device is available, you can use the following method. The high-content analysis is a system that combines a highly automated fluorescence microscope with image analysis software. Therefore, although the objectivity and high-throughput capability are lost, you can use fluorescence microscopy and any image analysis software, such as Image J, to evaluate ROS production.

### Problem 5

High-content analysis CQ1 is available only in a non-BSL room. Is it possible to take a plate with cultured human endothelial cells out of a BSL-2 room? (Step 10 in step-by-step method details)

### Potential solution

Before implementing this solution, be sure to check the rules of your facility and obtain permission from the facility manager if necessary. Complete prevention of scattering of the cell culture medium and sterilization of the outside of the plate may allow it to be taken out. After the wash in step 9 of “step-by-step method details” section, seal the wells of the 96-well culture plate with a seal having low light reflectivity. After confirming that all wells are completely sealed, wipe all over the outside of the plate with a disposable towel containing a sterilizer, such as hypochlorous acid. Then, set the plate into a pre-sterilized airtight box, and carry it from the BSL-2 room to the room where the CQ1 is installed. It is advisable to avoid people as much as possible during transport and to take the shortest route possible. Set the sealed plate into the CQ1, and capture the fluorescence images. After image capture, return the plate to the BSL-2 room as soon as possible.

## Resource availability

### Lead contact

Further information and requests for resources and reagents should be directed to and will be fulfilled by the lead contact, Minako Hoshi (minako.stella.hoshi.37@fbri.org).

### Materials availability

This study did not generate new unique reagents.

## Data Availability

This study did not generate datasets and not analyze code.
